# Management and outcomes of myocardial infarction in people with impaired kidney function in England

**DOI:** 10.1186/s12882-023-03377-x

**Published:** 2023-11-02

**Authors:** Jemima Scott, Patrick Bidulka, Dominic M. Taylor, Udaya Udayaraj, Fergus J. Caskey, Kate Birnie, John Deanfield, Mark de Belder, Spiros Denaxas, Clive Weston, David Adlam, Dorothea Nitsch

**Affiliations:** 1 Population Health Sciences, University of Bristol, Bristol, England; 2 Richard Bright Renal Service, North Bristol NHS Trust, Southmead Hospital, Bristol, England; 3 Department of Non-Communicable Disease Epidemiology, London School of Hygiene & Tropical Medicine, Keppel Street, London, England; 4 Oxford Kidney Unit, Churchill Hospital, Oxford, England; 5 Nuffield Department of Medicine, University of Oxford, Oxford, England; 6 National Institute for Cardiovascular Outcomes Research (NICOR), NHS Arden & Greater East Midlands Commissioning Support Unit, Leicester, England; 7 Institute of Cardiovascular Sciences, University College London, London, UK; 8 British Heart Foundation, Data Science Centre, London, UK; 9 University College London Hospitals Biomedical Research Centre, London, UK; 10 Glangwili General Hospital, Dolgwili Road, Carmarthen, Wales UK; 11 Department of Cardiovascular Sciences, University of Leicester, and NIHR Leicester Biomedical Research Centre, Leicester, UK

**Keywords:** CKD, Coronary angiography, Myocardial infarction, Percutaneous coronary intervention, Survival analysis

## Abstract

**Background:**

Acute myocardial infarction (AMI) causes significant mortality and morbidity in people with impaired kidney function. Previous observational research has demonstrated reduced use of invasive management strategies and inferior outcomes in this population. Studies from the USA have suggested that disparities in care have reduced over time. It is unclear whether these findings extend to Europe and the UK.

**Methods:**

Linked data from four national healthcare datasets were used to investigate management and outcomes of AMI by estimated glomerular filtration rate (eGFR) category in England. Multivariable logistic and Cox regression models compared management strategies and outcomes by eGFR category among people with kidney impairment hospitalised for AMI between 2015–2017.

**Results:**

In a cohort of 5 835 people, we found reduced odds of invasive management in people with eGFR < 60mls/min/1.73m^2^ compared with people with eGFR ≥ 60 when hospitalised for non-ST segment elevation MI (NSTEMI). The association between eGFR and odds of invasive management for ST-elevation MI (STEMI) varied depending on the availability of percutaneous coronary intervention. A graded association between mortality and eGFR category was demonstrated both in-hospital and after discharge for all people.

**Conclusions:**

In England, patients with reduced eGFR are less likely to receive invasive management compared to those with preserved eGFR. Disparities in care may however be decreasing over time, with the least difference seen in patients with STEMI managed via the primary percutaneous coronary intervention pathway. Reduced eGFR continues to be associated with worse outcomes after AMI.

**Supplementary Information:**

The online version contains supplementary material available at 10.1186/s12882-023-03377-x.

## Introduction

The prevalence of moderate to severe chronic kidney disease (CKD) is 11–13% in the general population [1], but 40% in those with acute myocardial infarction (AMI) [2–4]. The increased risk of AMI with CKD results from higher prevalence of traditional cardiovascular risk factors in addition to risk factors specific to CKD pathophysiology [5]. In the United States of America (USA) and Europe, in-hospital and post-discharge mortality following AMI is higher in the CKD population [6, 7]. Progressively worse outcomes are seen with increasing CKD severity [2].

Knowledge of the optimal management of AMI in people with CKD lags behind our understanding within the general population. People with CKD have been excluded from most randomized-controlled trials (RCTs) that have driven forward advances in AMI management over the past 50 years [8]. Evidence in this population is limited to observational data and small subgroup analyses of people with mild to moderate CKD who were included within relevant RCTs [9–12]. The accumulating evidence suggests that people with reduced estimated glomerular filtration rate (eGFR) do benefit from invasive management of AMI, despite increased risk of complications and poorer outcomes compared to those with normal kidney function [13–15]. Current USA and European cardiology guidelines now recommend invasive management for high-risk non-ST-elevation myocardial infarction (NSTEMI) and all ST-elevation myocardial infarction (STEMI) events independent of eGFR [16, 17].

Previous observational research has shown that people with reduced eGFR receive less aggressive AMI management than those with normal kidney function [3, 10, 11, 14, 18]. Despite evidence that disparities in care are falling over time, contemporary studies from the USA continue to demonstrate reduced rates of invasive management in patients with low eGFR [6, 19, 20]. Since the routine introduction of primary percutaneous coronary intervention (PCI) for STEMI in 2009, no studies from Europe or the UK have examined how this may have affected care for patients with reduced eGFR [21]. In England, the latest study relating to AMI in patients with kidney disease used data from 2004–2008 and was limited to NSTEMI [13]. To our knowledge, the association between eGFR and treatment of STEMI has not previously been examined using English data.

Prior research regarding AMI care for people with reduced eGFR in England is limited because granular data on eGFR and AMI are held on distinct healthcare registries. Reliance on a single dataset risks biasing results via misclassification of disease status. To maximise the utility of available data, we used multiple linked healthcare registries to provide an updated description of AMI care and outcomes for people with reduced eGFR in England. Dataset linkage has allowed a) the use of pre-admission rather than in-hospital creatinine readings to define eGFR category, giving a more accurate reflection of baseline kidney function [22], and b) the identification of AMI hospitalisations from more than one database, optimizing sensitivity in identifying these events [22, 23].

## Materials and methods

### Study design and data sources

This historical cohort study used data from clinical audit and routinely collected health records. Our cohort was defined using primary care data from the National Chronic Kidney Disease Audit (NCKDA) research database, and secondary care data from the Myocardial Ischaemia National Audit Project (MINAP) and Hospital Episode Statistics (HES) Admitted Patient Care (APC).

The NCKDA [24, 25] aimed to audit and improve primary care health services in England and Wales for people with CKD or CKD risk factors (Additional Table 1). The audit included 10% of English General Practices (approximately 1.7 million people with CKD or risk factors for CKD) who invested in audit software and volunteered to participate in the audit, and is now used as a research database to study long-term outcomes of this population [22, 24, 25]. The NCKDA collected complete historical patient-level data for eligible participants from general practices in two main cross-sectional extracts between 2014 and 2016. People who died between extracts, opted-out of data-sharing (person or practice-level), or people who changed GPs were excluded in the second extract. People included in the NCKDA were generally representative of the English population in terms of age and sex [24].

HES Admitted Patient Care data are collected to compensate hospitals for services provided by the NHS. In England, all hospitalisations funded by the NHS (approximately 99%) are captured by HES data [26]. Diagnoses during hospitalisation are recorded using International Classification of Diseases 10^th^ Edition (ICD-10) codes.

MINAP, part of the National Institute of Cardiovascular Outcomes Research (NICOR) audit and research programme, aims to audit all type 1 AMIs admitted to hospitals in England and Wales. Data are collected on patient characteristics, laboratory tests, comorbidities, processes of care, and treatment received during AMI hospitalisation [27, 28].

Office of National Statistics (ONS) data were linked to these primary and secondary care data to determine death dates [29] (Additional Table 2). The Index of Multiple Deprivation (IMD) patient-level data were linked as a proxy for socioeconomic status (SES) [30].

HES and MINAP-linked data were available up to 31 March 2017. Anyone with an AMI hospitalisation between the final NCKDA extract and the end of HES/MINAP linked data were included in the cohort. The ONS linked follow-up death data were available up to 15 September 2019.

### Study participants

We included people captured by the NCKDA research database with an AMI hospitalisation recorded in MINAP, HES, or both between 2015–2017, after the final NCKDA cross-sectional extract in which the person appeared [22]. We identified incident AMI hospitalisations and AMI subtypes (STEMI, NSTEMI) in HES using ICD-10 codes (Additional Table 3) recorded in the first diagnostic position of the first episode of the spell, and in MINAP using an algorithm which uses discharge diagnosis, cardiac marker levels, and electrocardiogram results (Additional Table 4). People with CKD risk factors, but no eGFR in the primary care record (*n* = 118), were excluded.

### Exposures

We calculated the baseline eGFR from the most recent serum creatinine value recorded in primary care prior to the index AMI hospitalisation using the MDRD equation [31]. We defined eGFR categories using the same cut-points KDIGO recommends for the definition of CKD stages: Category 1–2 (eGFR 60-120 mL/min/1.73m^2^), 3a (eGFR 45–59), 3b (eGFR 30–44), and 4–5 (eGFR 0–29) [32].

### Outcomes

Our primary outcomes were all-cause death during the first AMI hospitalisation recorded during the study period (the index AMI hospitalisation), and all-cause death during follow-up, for those who survived the index AMI hospitalisation. Variables used to define death date are described in Additional Table 5. Secondary outcomes were treatments received during hospitalisation: (1) Angiography and/or percutaneous coronary intervention (PCI), and (2) coronary artery bypass graft (CABG) (Additional Table 6). Other secondary outcomes, among survivors of the index AMI hospitalisation, were AMI re-admission and cardiovascular-specific death post-index AMI discharge.

### Covariables

Potential confounding variables available in our dataset were age at AMI hospitalisation (continuous), sex, ethnicity (white, other), IMD quintile, smoking status (non-smoker, ever smoker), receipt of dialysis or kidney transplant, prior AMI, and comorbidities including chronic obstructive pulmonary disease (COPD), type 2 diabetes mellitus (T2DM), heart failure, unstable angina, cerebrovascular disease, hypertension, and peripheral vascular disease. We defined these covariates using a combination of primary and secondary care data (Additional Table 7) [22]. We categorised each hospital centre which contributed patient-level data to this study into two main categories: (1) PCI always available and (2) PCI services not always available (Additional Table 8).

### Data analysis

We described baseline characteristics of the study population stratified by eGFR category. We used multivariable logistic regression to estimate the adjusted odds ratios comparing the odds of death during the index AMI hospitalisation (primary outcome) and the odds of invasive management (angiography and/or PCI, coronary artery bypass graft (CABG)) across eGFR categories. We also calculated predicted percentages from the adjusted logistic regression models using recycled predictions, since odds ratios can be misleading when the outcome is common [33]. We looked at these associations in the overall study population and stratified by AMI subtype (STEMI and NSTEMI). We tested for a linear trend in the association between eGFR category and the odds of receiving angiography and/or PCI using a likelihood ratio test.

We used Cox regression to investigate the association between eGFR category and outcomes post-index AMI hospitalisation among survivors, including all-cause mortality (primary outcome), cardiovascular-specific mortality, and AMI re-admission, after confirming the proportional hazard assumption using a global test on the Schoenfeld residuals over time. We first calculated crude rates for each outcome stratified by eGFR stage by dividing the number of outcome events by the total person-time study participants contributed following discharge from the index AMI hospitalisation. We reported these crude rates per 100 person-years. In our multivariable models, we specified a priori to adjust for age (continuous), sex, ethnicity, IMD quintile, COPD, T2DM, heart failure, and prior AMI as we anticipated these to be the most important confounders for this study population.

### Secondary/sensitivity analyses

We repeated the main analyses, stratifying by (1) centre type, to understand the impact of PCI availability on the association between eGFR category and the odds of receiving angiography and/or PCI; (2) and relevant comorbidities (prevalent T2DM and heart failure), since it is possible people with these comorbidities experience different management and outcomes compared with those without. We also repeated all main analyses after excluding people with prior AMI (*n* = 1,883) as previous coronary intervention may impact subsequent care.

### Missing data

We conducted a complete case analysis, excluding people with missing ethnicity and/or IMD data (*n* = 107). Discharge dates were missing in 19% of MINAP and 1% of HES records. We imputed missing discharge dates using the median number of days in-hospital from non-missing records (5 and 4 days in MINAP and HES, respectively) [22].

### Patient and public involvement

This study benefited from similar patient and public involvement as described in a related study [22]. The creation and maintenance of the NCKDA research database, including its record linkages and necessary section 251 permissions benefited from the support of the Kidney Care UK patient organisation (https://www.kidneycareuk.org/). Feedback from patient members of the UK Renal Registry Patient Council (https://renal.org/patients/patient-council) supported a further planned record linkage of renal and cardiac data.

## Results

### Study population and baseline characteristics

**Fig. 1:**
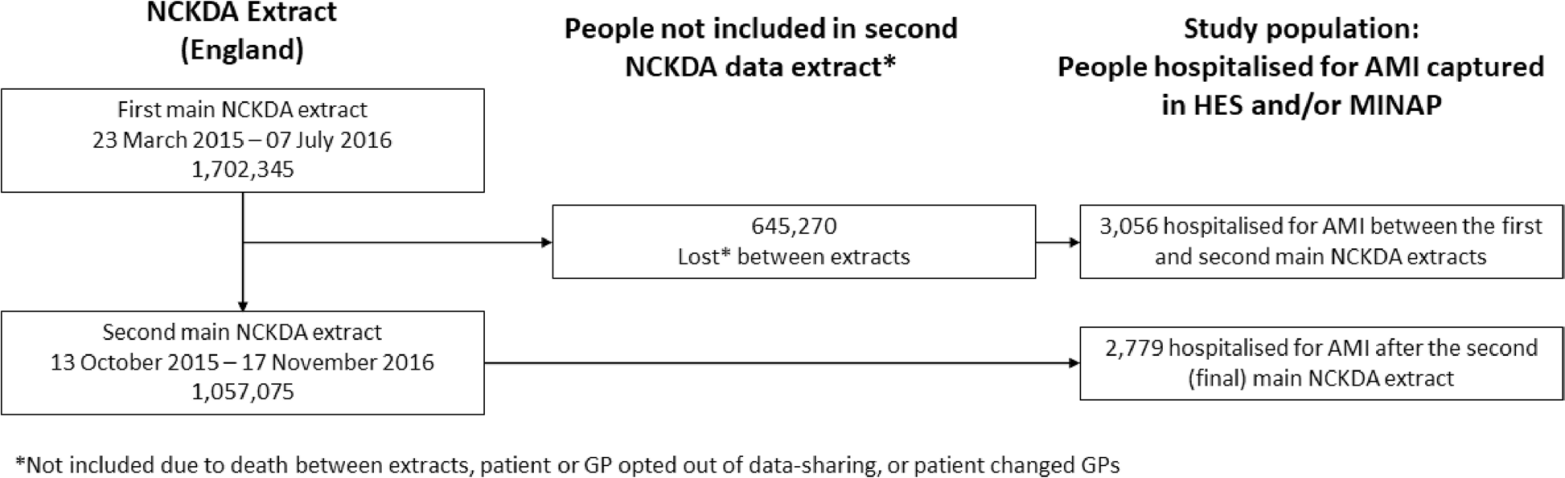
Flow diagram illustrating the study population hospitalised for AMI derived from the NCKDA research database

A total of 5 835 individuals who were included in the NCKDA and experienced at least one incident AMI hospitalisation captured in HES and/or MINAP were included in this study (Fig. 1). The median time between the most recent eGFR recorded in primary care and the index AMI hospitalisation was 0.97 years (interquartile range (IQR) 0.60 to 1.63) (Additional Fig. 1).

Of the 5 835 people hospitalised for AMI during the study period, 2,260 (39%) had eGFR category 3–5 as their latest primary care record of kidney function (Table 1). People with eGFR category 3b were oldest on average (82 years) and had the highest proportion of females (50%) compared with other eGFR categories. The most prevalent comorbidity was hypertension (60% overall), followed by type two diabetes mellitus (34%), and angina (25%). People with incomplete covariate data (*n* = 107) are described in Additional Table 9.

**Table 1:** Baseline characteristics of people included in the NCKDA with an AMI hospitalisation recorded in MINAP, HES, or both

eGFR category	1–2	3a	3b	4–5	Total
	***N* = 3,574**	***N* = 1,193**	***N* = 721**	***N* = 347**	**5,835**
**Age (years) at AMI admission, mean (SD)**	70 (13)	79 (10)	82 (9)	79 (12)	74 (13)
**Age group at AMI admission, years**					
< 50	249 (7)	15 (1)	1 (0)	9 (3)	274 (5)
50–64	976 (27)	83 (7)	36 (5)	35 (10)	1,130 (19)
65–79	1,443 (40)	477 (40)	213 (30)	104 (30)	2,237 (38)
80 +	906 (25)	618 (52)	471 (65)	199 (57)	2,194 (38)
**Female**	1,226 (34)	564 (47)	361 (50)	142 (41)	2,293 (39)
**Ethnicity**					
White	3,288 (92)	1,131 (95)	686 (95)	317 (91)	5,422 (93)
Other	286 (8)	62 (5)	35 (5)	30 (9)	413 (7)
**IMD quintile**					
1 (least deprived)	631 (18)	226 (19)	136 (19)	59 (17)	1,052 (18)
2	736 (21)	274 (23)	154 (21)	68 (20)	1,232 (21)
3	793 (22)	281 (24)	167 (23)	72 (21)	1,313 (23)
4	822 (23)	229 (19)	159 (22)	83 (24)	1,293 (22)
5 (most deprived)	592 (17)	183 (15)	105 (15)	65 (19)	945 (16)
**History of dialysis in primary care data**					
Peritoneal dialysis	0 (0)	0 (0)	0 (0)	10 (3)	10 (0)
Haemodialysis	0 (0)	0 (0)	0 (0)	21 (6)	21 (0)
Renal dialysis, unspecified	0 (0)	0 (0)	0 (0)	7 (2)	7 (0)
**History of kidney transplant in primary care data**	2 (0)	0 (0)	3 (0)	15 (4)	20 (0)
**Comorbidities**					
Angina	770 (22)	332 (28)	228 (32)	134 (39)	1,464 (25)
Cerebrovascular disease	319 (9)	159 (13)	126 (17)	68 (20)	672 (12)
COPD	422 (12)	184 (15)	147 (20)	48 (14)	801 (14)
Type 2 diabetes mellitus	1,086 (30)	408 (34)	307 (43)	205 (59)	2,006 (34)
Heart failure	287 (8)	191 (16)	174 (24)	103 (30)	755 (13)
Hypertension	1,942 (54)	765 (64)	509 (71)	276 (80)	3,492 (60)
History of acute myocardial infarction	650 (18)	314 (26)	205 (28)	128 (37)	1,297 (22)
Peripheral vascular disease	179 (5)	90 (8)	59 (8)	39 (11)	367 (6)
**Smoking status**					
Non-smoker	1,715 (48)	575 (48)	345 (48)	170 (49)	2,805 (48)
Ever-smoker	1,859 (52)	618 (52)	376 (52)	177 (51)	3,030 (52)

*n* = (col %) unless specified otherwise

### Death during AMI hospitalisation

Overall, 907 people (16%) died during the index AMI hospitalisation. The crude proportion who died was greatest among people in eGFR categories 4–5 (27%) and lowest among people in category 1–2 (11%) (Table 2).

**Table 2:** Death during the index AMI hospitalisation, overall and stratified by AMI subtype

Subgroup	eGFR category	Deaths, n (row %)	Total number of people	Age and sex adjusted, OR (95% CI)	Adjusted^a^, OR (95% CI)
**Overall**	1–2	399 (11)	3,574	1	1
	3a	231 (19)	1,193	1.30 (1.08–1.56)	1.28 (1.06–1.54)
	3b	183 (25)	721	1.60 (1.30–1.97)	1.51 (1.22–1.87)
	4–5	94 (27)	347	2.00 (1.53–2.62)	1.80 (1.37–2.38)
**AMI subtype**					
**STEMI**	1–2	174 (13)	1,339	1	1
	3a	75 (24)	314	1.34 (0.97–1.86)	1.26 (0.91–1.76)
	3b	55 (35)	159	1.81 (1.20–2.71)	1.63 (1.09–2.43)
	4–5	22 (31)	70	1.93 (1.08–3.46)	1.31 (0.73–2.38)
**NSTEMI**	1–2	225 (10)	2,235	1	1
	3a	156 (18)	879	1.35 (1.07–1.70)	1.33 (1.06–1.68)
	3b	128 (23)	562	1.64 (1.27–2.13)	1.56 (1.20–2.02)
	4–5	72 (26)	277	2.20 (1.58–3.07)	2.07 (1.50–2.86)

^a^Adjusted for age (continuous), sex, IMD quintile, ethnicity (white or other), and history of T2DM, heart failure, COPD, and previous AMI

After adjustment, we found that people in eGFR categories 3a, 3b and 4–5 had greater odds of death compared with people in category 1–2 (adjusted OR 1.28 (95% CI 1.06–1.54), 1.51 (95% CI 1.22–1.87), and 1.80 (95% CI 1.37–2.38), respectively) (Table 2). When stratifying by AMI subtype, we found similarly increased odds of death during AMI hospitalisation for people in eGFR categories 3a, 3b and 4–5 compared with category 1–2 among people with NSTEMI and STEMI (although 95% CI overlap the null estimate when comparing eGFR category 3a and 4–5 with category 1–2 for the STEMI subgroup). The predicted percents for death during AMI hospitalisation also showed a higher percent of people dying with eGFR stages 3b and 4–5 compared with eGFR stages 1–2 (Additional Table 10).

### Death post-AMI hospitalisation

Among people who survived their first AMI hospitalisation, we observed increasing rates of subsequent death with worsening baseline kidney function, during a mean follow-up of 2.4 years. People in eGFR category 1–2 had a crude rate of death post-AMI hospitalisation of 8.30 per 100 person-years (PY) (95% CI 7.70–8.95), while people in category 4–5 had a crude rate of death of 54.33 per 100 PY (95% CI 47.04–62.76) (Table 3). There was no evidence that the hazards were not proportional over time (*p* = 0.18).

**Table 3:** Death post-AMI hospitalisation, among people who survive the index AMI hospitalisation, overall and stratified by AMI subtype

Subgroup	eGFR category	Deaths, *n* =	Rate per 100 person-years	Age and sex adjusted, Hazard Ratio (HR) (95% CI)	Adjusted^a^, HR (95% CI)
**Overall**	1–2	675	8.30 (7.70–8.95)	1	1
	3a	359	16.94 (15.23–18.78)	1.19 (1.04–1.36)	1.10 (0.96–1.26)
	3b	298	30.37 (27.11–34.02)	1.63 (1.41–1.88)	1.40 (1.21–1.62)
	4–5	185	54.33 (47.04–62.76)	3.12 (2.64–3.69)	2.57 (2.16–3.05)
**AMI subtype**					
**STEMI**	1–2	187	6.08 (5.26–7.01)	1	1
	3a	71	13.37 (10.59–16.87)	1.18 (0.89–1.56)	1.20 (0.90–1.59)
	3b	50	26.22 (19.88–34.60)	1.52 (1.09–2.12)	1.33 (0.94–1.86)
	4–5	29	45.82 (31.84–65.94)	3.56 (2.38–5.32)	3.47 (2.29–5.26)
**NSTEMI**	1–2	488	9.65 (8.83–10.55)	1	1
	3a	288	18.13 (16.15–20.35)	1.17 (1.01–1.36)	1.08 (0.93–1.26)
	3b	248	31.37 (27.70–35.53)	1.62 (1.38–1.90)	1.41 (1.20–1.65)
	4–5	156	56.28 (48.10–65.84)	2.99 (2.48–3.60)	2.49 (2.06–3.02)

^a^Adjusted for age (continuous), sex, IMD quintile, ethnicity (white or other), and history of T2DM, heart failure, COPD, and previous AMI

After adjusting for pre-specified confounders, we observed increased hazards of death among people in eGFR categories 3b and 4–5, compared with people in category 1–2 (adjusted HR 1.40 (95% CI 1.21–1.62) and 2.57 (95% CI 2.16–3.05), respectively). Hazards were similarly greater among people in eGFR categories 3b and 4–5, compared with people in category 1–2 when stratifying by AMI subtype (Table 3).

### Processes of care during AMI hospitalisation

Overall, the crude proportion of people who received angiography and/or PCI during their first AMI hospitalisation in the study period ranged from 64% among people in eGFR category 1–2 to 33% among people in category 4–5 (Table 4).

**Table 4:** Processes of care (angiography and/or PCI) associated with eGFR at baseline during the index AMI hospitalisation, overall and stratified by AMI subtype

Outcome/Subgroup	eGFR category	N^b^ (row %)	Total number of people	Age and sex adjusted, OR (95% CI)	Adjusted^a^, OR (95% CI)
**Angiography and/or PCI**					
**Overall**	1–2	2,280 (64)	3,574	1	1
	3a	602 (50)	1,193	1.01 (0.87–1.17)	1.08 (0.93–1.26)
	3b	259 (36)	721	0.66 (0.55–0.79)	0.76 (0.63–0.92)
	4–5	117 (33)	347	0.44 (0.34–0.57)	0.55 (0.42–0.71)
**AMI subtype**					
**STEMI**	1–2	1,106 (83)	1,339	1	1
	3a	223 (71)	314	0.96 (0.71–1.30)	1.05 (0.76–1.44)
	3b	90 (57)	159	0.68 (0.46–1.00)	0.78 (0.53–1.15)
	4–5	41 (59)	70	0.53 (0.29–0.96)	0.86 (0.47–1.55)
**NSTEMI**	1–2	1,174 (53)	2,235	1	1
	3a	379 (43)	879	1.15 (0.97–1.36)	1.21 (1.01–1.44)
	3b	169 (30)	562	0.74 (0.60–0.91)	0.82 (0.66–1.02)
	4–5	76 (27)	277	0.50 (0.37–0.67)	0.54 (0.40–0.73)
**CABG**					
**Overall**	1–2	83 (2)	3,574	1	1
	3a	33 (3)	1,193	1.68 (1.08–2.59)	1.64 (1.05–2.55)
	3b	9 (1)	721	0.84 (0.41–1.73)	0.77 (0.37–1.60)
	4–5	6 (1)	347	0.99 (0.43–2.32)	0.80 (0.34–1.91)

^a^Adjusted for age (continuous), sex, IMD quintile, ethnicity (white or other), and history of T2DM, heart failure, COPD, and previous AMI

^b^n Is the number of people receiving angiography and/or PCI

In our adjusted analysis, we observed that people in eGFR categories 3b and 4–5 had lower odds of receiving angiography and/or PCI compared with people in category 1–2 (adjusted OR 0.76 (95% CI 0.63–0.92) and 0.55 (95% CI 0.42–0.71), respectively). The predicted percents also showed a lower percentage of people receiving angiography and/or PCI with eGFR stages 3b and 4–5 compared with eGFR stages 1–2 (Additional Table 10). When stratifying by AMI subtype, the association persisted among people with NSTEMI. People in eGFR category 4–5 had lower odds of receiving angiography and/or PCI compared with people in category 1–2 (adjusted OR 0.54 (95% CI 0.40–0.73). There was no evidence for a trend in association (*p* = 0.32) (Table 4).

We did not see evidence of an association between eGFR and receiving CABG after adjusting for pre-specified confounders, although our analyses were limited by low numbers of CABG recipients (Table 4).

### Other outcomes

The crude rate of CVD-specific death ranged from 3.74 per 100 PY (95% CI 3.34–4.18) in people in eGFR category 1–2 to 23.20 per 100 PY (95% CI 18.61–28.93) in people in category 4–5 among those discharged alive from the index AMI hospitalisation. There was evidence of increased hazards of CVD-specific death among people in eGFR categories 3b and 4–5 versus people in category 1–2 (Additional Table 11).

Crude rates of AMI re-hospitalisation among people discharged alive after their first AMI hospitalisation in the study period ranged from 22.50 per 100 PY (95% CI 20.15–25.12) among people in eGFR category 3b to 45.04 per 100 PY (95% CI 32.77–61.89) among people in category 4–5. There was no evidence of increased hazards of AMI re-hospitalisation with worsening eGFR after adjustment for potential confounders (Additional Table 11).

### Secondary/sensitivity analyses

When stratifying by availability of PCI services, we observed an attenuation of the relative odds of death during the index AMI hospitalisation for people in eGFR categories 3a (adjusted OR 0.84, 95% CI 0.58–1.22) and 3b (adjusted OR 1.23 (95% CI 0.82–1.85)) versus people in category 1–2 in centres where PCI is always available (Additional Table 12). Relative hazards of death post-AMI hospitalisation among people who survived were similarly greater for people with worsening eGFR when stratifying by PCI service availability (Additional Table 13).

The odds of receiving angiography and/or PCI during the index AMI hospitalisation were similarly lower among people in eGFR categories 3b and 4–5 compared with people in category 1–2 both in centres with and without constant PCI availability. When restricting to STEMI hospitalisations, there was no association between eGFR category and odds of receiving angiography and/or PCI in the centres where PCI is always available. However, there were lower odds of people in eGFR categories 3a, 3b and 4–5 receiving angiography and/or PCI compared with people in category 1–2 when restricting to centres where PCI is available sometimes or not at all (Additional Table 14).

We observed no substantial changes to our results when excluding people with a history of AMI (Additional Table 15), nor when stratifying by prevalent T2DM status (Additional Figs. 3 and 4). However, there was no evidence of an association between eGFR category and receipt of angiography and/or PCI among people with recorded prevalent heart failure (Additional Fig. 2).

## Discussion

In this analysis of 5 835 AMI hospitalisations from linked primary and secondary care multi-disease registries in England, odds of death both in-hospital and post-discharge were significantly higher in people with a pre-admission eGFR < 60mls/min/1.73m^2^, compared to those with an eGFR ≥ 60. We demonstrated a progressive reduction in the odds of receiving angiography and/or PCI for NSTEMI with reducing eGFR, independent of the availability of PCI services. In contrast, in people with STEMI, we found no association between eGFR category and the odds of invasive management in the population overall. In centres where PCI was not always available however, reduced use of angiography and PCI extended to those with STEMI, suggesting an opportunity to improve outcomes of patients with impaired renal function by better access to specialised centers with primary PCI services.

Reducing eGFR was associated with a progressive increase in the odds of death following all AMI events both within hospital and post-discharge. Inferior mortality outcomes have been reported previously amongst people with kidney disease, with the poorest survival in those with the lowest eGFRs [10, 14, 18]. People with low eGFR have a higher baseline mortality risk prior to AMI, and are more likely to experience complications relating to both the AMI event and its treatment [2, 6, 7]. Increased deaths amongst people with reduced eGFR could be due to residual confounding from severity of comorbidities or unmeasured factors such as frailty. Reduced invasive AMI management has also been suggested to contribute to these worse outcomes [13, 14, 34, 35].

We demonstrated an inverse association between eGFR category and the odds of invasive management after NSTEMI. Reduced use of angiography and revascularisation in people with kidney impairment has been described previously, and may relate to concerns about contrast-induced nephropathy and bleeding risks, or therapeutic nihilism [3, 35, 36]. A study from the USA has however shown narrowing of this treatment gap, with the greatest increase in use of invasive management in those with the worst kidney function [6]. Comparison of our data with that from a study of NSTEMI management in England in 2004–2008 suggests a similar relative increase in the use of angiography in the lowest eGFR categories [13].

We found that reduced eGFR is associated with lower odds of invasive management in people with NSTEMI but not in those with STEMI. Possible explanations for these differences include a) PCI in STEMI is time-critical and clinicians may not have time to review blood results and/or b) clinicians may consider the benefits of PCI in STEMI to outweigh the risks posed to kidney function. It is possible that our small sample size underlies this lack of association. A large study of AMI management and outcomes in the USA demonstrated reduced use of angiography after STEMI in people with CKD in 2007–8, with attenuation of these differences in 2014–15, following the routine introduction of primary PCI [6]. This correlates with our findings of an association between eGFR and the receipt of angiography which is limited to centres that do not always offer PCI. Reduced use of angiography in these centres may reflect reluctance by clinicians to intervene in frail and complex patients, or transfer to centres offering primary PCI. The lack of association between eGFR and odds of invasive management after AMI (any) in people with heart failure may simply reflect poor diagnosis and recording of heart failure amongst patients with kidney disease [37].

There are some limitations to consider. First, although MINAP is designed as an audit of type one AMI, we were unable to exclude type two AMIs from our analyses. These events may occur more frequently in people with low eGFR than without [14]. Similarly, we may have assigned incorrect eGFR categories to patients experiencing AKI either prior to, or at the time of, admission with AMI. Secondly, we were unable to risk stratify our AMI cohort. Reduced eGFR is associated with greater cardiac risk however, so differences in risk are unlikely to explain our findings. Thirdly, the competing risk of death may bias our effect estimates when investigating receipt of invasive management. The number of people dying within the decision-making timeframe are, however, likely to be small. Fourth, residual confounding is likely to affect our results, for example severity of comorbidities and pharmacological management. Fifth, we acknowledge the study population is selected from 10% of GPs in England who self-selected to take part in the NCKDA, and may not be representative of the English population in terms of ethnicity and standard of primary care [24]. Finally, overestimation of baseline kidney function is also possible, since the median time between the most recent serum creatinine test and the index AMI hospitalisation was approximately one year during which time kidney function may have worsened.

This study adds evidence from England to existing international research demonstrating disparities in AMI care and outcomes between those with and without reduced eGFR. Further research is needed to understand why eGFR influences receipt of AMI management and explore whether differences in AMI care represent appropriate risk stratification of people with reduced eGFR, or inequitable access to effective management. Understanding these treatment disparities will enable interventions to be appropriately allocated to optimize care and outcomes for the growing global CKD population.

### Supplementary Information

**Additional file 1: Additional table 1.** Risk factors for CKD which fulfilled inclusion criteria for the NCKDA. **Additional table 2.** Details on linkages between study datasets. **Additional table 3.** ICD-10 codes for AMI identified in HES*. **Additional table 4.** CALIBER definition of AMI subtypes (STEMI, NSTEMI) using MINAP data.*. **Additional table 5.** Variables used to define death in-hospital and post-AMI discharge*. **Additional table 6.** Definitions for processes of AMI care in MINAP and HES datasets. **Additional table 7.** Details on data sources for covariates*. **Additional table 8.** Categorisation of PCI services available at each hospital in England for this study, derived from categorisation of PCI services available at each hospital in England by MINAP researchers. **Additional table 9.** Baseline characteristics of people with incomplete covariate data who are dropped from the complete case analysis (*N*=107). **Additional table 10.** Adjusted predicted percents for dying during the index AMI hospitalisation, and for receiving angiography and/or PCI during the index AMI hospitalisation, stratified by eGFR stage. **Additional table 11.** Other outcomes during the index AMI hospitalisation and post-AMI hospitalisation. **Additional table 12.** Death during the index AMI hospitalisation stratified by centre type. **Additional table 13.** Death post-AMI hospitalisation discharge, among people who survive the first AMI hospitalisation in the study period, stratified by AMI subtype and centre type. **Additional table 14.** Processes of care (angiography and/or PCI) associated with eGFR stage at baseline during the index AMI hospitalisation stratified by centre type in the overall population, and among people with STEMI hospitalisation only. **Additional table 15.** Processes of care (angiography and/or PCI) during the index AMI hospitalisation restricted to people with no previous AMI hospitalisation. **Additional table 16.** MDRD Study equation. **Additional figure 1.** Histogram describing the time between the most recent eGFR recorded in primary care (used to define baseline kidney function) and the index AMI hospitalisation. **Additional figure 2.** Processes of care (angiography and/or PCI) associated with eGFR stage at baseline during the index AMI hospitalisation stratified by prevalent type 2 diabetes mellitus (T2DM) and prevalent heart failure status. **Additional figure 3.** In-hospital death associated with eGFR stage at baseline during the index AMI hospitalisation stratified by prevalent type 2 diabetes mellitus (T2DM) and prevalent heart failure status. **Additional figure 4.** Death post-AMI discharge associated with eGFR stage at baseline during the index AMI hospitalisation stratified by prevalent type 2 diabetes mellitus (T2DM) and prevalent heart failure status.

## Data Availability

The data that support the findings of this study are available from the NCKDA and MINAP but restrictions apply to the availability of these data, which were used under license for the current study, and so are not publicly available. For further information contact Jemima.Scott@bristol.ac.uk.

## Abbreviations

AMI: Acute myocardial infarction
CABG: Coronary artery bypass graft
CKD: Chronic kidney disease
COPD: Chronic obstructive pulmonary disease
eGFR: Estimated glomerular filtration rate
HES-APC: Hospital Episode Statistics—Admitted Patient Care
ICD-10: International Statistical Classification of Diseases and Related Health Problems—10th Revision
IMD: Indices of multiple deprivation
KDIGO: Kidney Disease Improving Global Outcomes
MDRD: Modification of Diet in Renal Disease
MINAP: Myocardial Ischaemia National Audit Project
NCKDA: National Chronic Kidney Disease Audit
NICOR: National Institute for Cardiovascular Outcomes Research
NSTEMI: Non ST-elevation myocardial infarction
ONS: Office for National Statistics
PCI: Percutaneous coronary intervention
RCT: Randomised controlled trial
STEMI: ST-elevation myocardial infarction
T2DM: Type two diabetes mellitus
