# Supplementary material for: Management and outcomes of myocardial infarction in people with impaired kidney function in England
Source: BMC Nephrol. 2023 Nov 2;24:325. doi: 10.1186/s12882-023-03377-x (PMC10623815; doi:10.1186/s12882-023-03377-x)
Supplement: Supplementary file 1 — Additional file 1: Additional table 1. Risk factors for CKD which fulfilled inclusion criteria for the NCKDA. Additional table 2. Details on linkages between study datasets. Additional table 3. ICD-10 codes for AMI identified in HES*. Additional table 4. CALIBER definition of AMI subtypes (STEMI, NSTEMI) using MINAP data.*. Additional table 5. Variables used to define death in-hospital and post-AMI discharge*. Additional table 6. Definitions for processes of AMI care in MINAP and HES datasets. Additional table 7. Details on data sources for covariates*. Additional table 8. Categorisation of PCI services available at each hospital in England for this study, derived from categorisation of PCI services available at each hospital in England by MINAP researchers. Additional table 9. Baseline characteristics of people with incomplete covariate data who are dropped from the complete case analysis (N=107). Additional table 10. Adjusted predicted percents for dying during the index AMI hospitalisation, and for receiving angiography and/or PCI during the index AMI hospitalisation, stratified by eGFR stage. Additional table 11. Other outcomes during the index AMI hospitalisation and post-AMI hospitalisation. Additional table 12. Death during the index AMI hospitalisation stratified by centre type. Additional table 13. Death post-AMI hospitalisation discharge, among people who survive the first AMI hospitalisation in the study period, stratified by AMI subtype and centre type. Additional table 14. Processes of care (angiography and/or PCI) associated with eGFR stage at baseline during the index AMI hospitalisation stratified by centre type in the overall population, and among people with STEMI hospitalisation only. Additional table 15. Processes of care (angiography and/or PCI) during the index AMI hospitalisation restricted to people with no previous AMI hospitalisation. Additional table 16. MDRD Study equation. Additional figure 1. Histogram describing the time between the mo [file 12882_2023_3377_MOESM1_ESM.doc]

**Additional materials**

Contents

[**Additional table 1:** Risk factors for CKD which fulfilled inclusion criteria for the NCKDA 4](#__RefHeading___Toc144297724)

[**Additional table 2:** Details on linkages between study datasets 5](#__RefHeading___Toc144297725)

[**Additional table 3:** ICD-10 codes for AMI identified in HES* 6](#__RefHeading___Toc144297726)

[**Additional table 4:** CALIBER definition of AMI subtypes (STEMI, NSTEMI) using MINAP data.* 7](#__RefHeading___Toc144297727)

[**Additional table 5:** Variables used to define death in-hospital and post-AMI discharge.* 8](#__RefHeading___Toc144297728)

[**Additional table 6:** Definitions for processes of AMI care in MINAP and HES datasets 9](#__RefHeading___Toc144297729)

[**Additional table 7:** Details on data sources for covariates.* 10](#__RefHeading___Toc144297730)

[**Additional table 8:** Categorisation of PCI services available at each hospital in England for this study, derived from categorisation of PCI services available at each hospital in England by MINAP researchers 11](#__RefHeading___Toc144297731)

[**Additional table 9:** Baseline characteristics of people with incomplete covariate data who are dropped from the complete case analysis (N=107) 12](#__RefHeading___Toc144297732)

### **Additional table 10:** Adjusted predicted percents for dying during the index AMI hospitalisation, and for receiving angiography and/or PCI during the index AMI hospitalisation, stratified by eGFR stage……………………………………………………………………………………………………………………..13

[**Additional table 11:** Other outcomes during the index AMI hospitalisation and post-AMI hospitalisation. 14](#__RefHeading___Toc144297733)

[**Additional table 12:** Death during the index AMI hospitalisation stratified by centre type 15](#__RefHeading___Toc144297734)

[**Additional table 13:** Death post-AMI hospitalisation discharge, among people who survive the first AMI hospitalisation in the study period, stratified by AMI subtype and centre type 16](#__RefHeading___Toc144297735)

[**Additional table 14:** Processes of care (angiography and/or PCI) associated with eGFR stage at baseline during the index AMI hospitalisation stratified by centre type in the overall population, and among people with STEMI hospitalisation only 17](#__RefHeading___Toc144297736)

[**Additional table 15:** Processes of care (angiography and/or PCI) during the index AMI hospitalisation restricted to people with no previous AMI hospitalisation 18](#__RefHeading___Toc144297737)

**Additional table 16:** MDRD Study equation……………………………………………………………………………………………………………………………………………….19

[**Additional figure 1:** Histogram describing the time between the most recent eGFR recorded in primary care (used to define baseline kidney function) and the index AMI hospitalisation 19](#__RefHeading___Toc144297738)

[**Additional figure 2:** Processes of care (angiography and/or PCI) associated with eGFR stage at baseline during the index AMI hospitalisation stratified by prevalent type 2 diabetes mellitus (T2DM) and prevalent heart failure status 20](#__RefHeading___Toc144297739)

[**Additional figure 3:** In-hospital death associated with eGFR stage at baseline during the index AMI hospitalisation stratified by prevalent type 2 diabetes mellitus (T2DM) and prevalent heart failure status 21](#__RefHeading___Toc144297740)

[**Additional figure 4:** Death post-AMI discharge associated with eGFR stage at baseline during the index AMI hospitalisation stratified by prevalent type 2 diabetes mellitus (T2DM) and prevalent heart failure status 22](#__RefHeading___Toc144297741)

### **Additional table 1:** Risk factors for CKD which fulfilled inclusion criteria for the NCKDA(1)

| **Risk factors for CKD** |
| --- |
| - Relevant cardiovascular disease - Diabetes mellitus - Hypertension - Connective tissue disorders - Prostatic disease - Kidney stones - Previous AKI - Family history of CKD - Previous prescriptions for kidney-damaging medications such as lithium or calcineurin inhibitors |

### **Additional table 2:** Details on linkages between study datasets

| **Dataset** | **Linkage details** |
| --- | --- |
| HES/ONS | HES/ONS data were linked by NHS Digital using NHS-numbers provided by the NCKDA study team and pseudonymised data were incorporated into the NCKDA research database. |
| MINAP | MINAP data were linked by NICOR to NCKDA data as the trusted third provider using NHS numbers; pseudonymised data were provided to the research team for analysis. |

### **Additional table 3:** ICD-10 codes for AMI identified in HES*

| **AMI subtype** | **ICD-10 codes** |
| --- | --- |
| Overall | I21, I22, I23 |
| STEMI | I21.0, I21.1, I21.2, I21.3, I22.0, I22.1, I22.8 |
| NSTEMI | I21.4, I21.9, I22.9 |
| Missing | I23 |

AMI: acute myocardial infarction; HES: Hospital Episode Statistics; ICD-10: International Classification of Diseases 10^th^ Edition; STEMI: ST elevated myocardial infarction; NSTEMI: Non-ST elevated myocardial infarction

*Table adapted from previous work: Bidulka P, Scott J, Taylor DM, et al. Impact of chronic kidney disease on case ascertainment for hospitalised acute myocardial infarction: an English cohort study. BMJ Open 2022;12:e057909. doi: 10.1136/bmjopen-2021-057909

### **Additional table 4:** CALIBER definition of AMI subtypes (STEMI, NSTEMI) using MINAP data.*

|  | **MINAP variable** | | |
| --- | --- | --- | --- |
| **AMI subtype** | **Discharge diagnosis** | **Markers elevated?** | **ECG result** |
| Other | Threatened MI, Chest pain uncertain cause, MI unconfirmed, other diagnosis | - | - |
| STEMI | STEMI | Raised or missing | ST elevation, LBBB, or ST elevation |
|  | NSTEMI/Troponin positive ACS | Raised or missing | ST elevation |
|  | ACS troponin negative | Raised | ST elevation |
|  | ACS troponin unspecified | Raised | ST elevation |
| NSTEMI | NSTEMI/Troponin positive ACS | Raised or missing | ST depression, T wave changes only, other abnormality, Normal ECG, or LBBB |
|  | ACS troponin negative | Raised | LBBB, ST depression, T wave changes only, Other abnormality, normal ECG, or missing |
|  | ACS troponin unspecified | Raised | LBBB, ST depression, T wave changes only, Other abnormality, normal ECG, or missing |
| Unstable angina | *Any remaining hospitalisations not assigned as STEMI, NSTEMI, or other diagnosis | | |
|  |  |  |  |
| ACS: acute coronary syndrome, AMI: acute myocardial infarction, ECG: electrocardiogram, LBBB: left bundle branch block, MI: myocardial infarction, MINAP: Myocardial Ischaemia National Audit Project, NSTEMI: Non ST-elevation myocardial infarction, STEMI: ST-elevation myocardial infarction | | | |

*Table adapted from previous work: Bidulka P, Scott J, Taylor DM, et al. Impact of chronic kidney disease on case ascertainment for hospitalised acute myocardial infarction: an English cohort study. *BMJ Open* 2022;12:e057909. doi: 10.1136/bmjopen-2021-057909

### **Additional table 5:** Variables used to define death in-hospital and post-AMI discharge.*

| **Data source** | **Variables to define in-hospital death** | **Variables to define death post-AMI discharge (in those who survive first AMI hospitalisation)** |
| --- | --- | --- |
| ONS | Date of death (falling on or in between first AMI admission and discharge dates) | Date of death (falling after first AMI discharge date) |
| MINAP | Reason for no angiography, no intervention, admission ward, discharge destination | - |
| HES | Discharge location, discharge method | - |
|  |  |  |
| AMI: acute myocardial infarction, HES: Hospital Episode Statistics, MINAP: Myocardial Ischaemia National Audit Project, ONS: Office of National Statistics | | |

*Table adapted from previous work: Bidulka P, Scott J, Taylor DM, et al. Impact of chronic kidney disease on case ascertainment for hospitalised acute myocardial infarction: an English cohort study. *BMJ Open* 2022;12:e057909. doi: 10.1136/bmjopen-2021-057909

### **Additional table 6:** Definitions for processes of AMI care in MINAP and HES datasets

| **Process of care** | **MINAP definition** | **HES definition** |
| --- | --- | --- |
| Angiography | Angiography defined using data from the following variables:   - Interventional hospital procedure - Coronary angiography - Why no angiography - Procedure performed - Additional reperfusion treatment - Why no intervention - Coronary intervention | Angiography defined by searching the opertn_XX variables (XX=01-24) using the following OPCS codes:   - K63 |
| PCI | PCI defined using data from the following variables:   - Why no intervention - Coronary intervention - Interventional hospital procedure - Procedure performed at admission - Initial reperfusion treatment - Additional reperfusion treatment | PCI defined by searching the opertn_XX variables (XX=01-24) using the following OPCS codes:   - K49 - K50 - K75 |
| CABG | CABG defined using data from the following variables:   - Why no intervention - Coronary intervention - Interventional hospital procedure | CABG defined by searching the opertn_XX variables (XX=01-24) using the following OPCS codes:   - K40 - K41 - K42 - K43 - K44 - K45 - K46 |

### **Additional table 7:** Details on data sources for covariates.*

| **Category** | **Covariate** | **Data source** |
| --- | --- | --- |
| Sociodemographic and lifestyle variables | Age at AMI admission | NCKDA |
|  | Sex | NCKDA |
|  | IMD quintiles | NCKDA |
|  | Smoking status | NCKDA, MINAP |
| Comorbidities | Unstable angina | MINAP and HES |
|  | Cerebrovascular disease | MINAP and HES |
|  | COPD | MINAP and HES |
|  | Diabetes mellitus | MINAP and HES |
|  | Heart failure | MINAP and HES |
|  | Hypertension | NCKDA, MINAP, and HES |
|  | Previous myocardial infarction | MINAP, and HES |
|  | Peripheral vascular disease | NCKDA, MINAP, and HES |
|  | Dialysis | NCKDA |
|  | Kidney transplant | NCKDA |
|  |  |  |
| AMI: Acute Myocardial Infarction, COPD: Chronic Obstructive Pulmonary Disease, HES: Hospital Episode Statistics, IMD: Index of Multiple Deprivation, NCKDA: National Chronic Kidney Disease Audit, MINAP: Myocardial Ischaemia National Audit Project | | |

*Table adapted from previous work: Bidulka P, Scott J, Taylor DM, et alImpact of chronic kidney disease on case ascertainment for hospitalised acute myocardial infarction: an English cohort studyBMJ Open 2022;12:e057909. doi: 10.1136/bmjopen-2021-057909

### **Additional table 8:** Categorisation of PCI services available at each hospital in England for this study, derived from categorisation of PCI services available at each hospital in England by MINAP researchers

| **PCI services categorised for this study** | **Original PCI services categorisation provided by MINAP researchers** |
| --- | --- |
| PCI services available sometimes or not at all | no PCI |
| PCI services available 24/7 | PCI 24/7, 365 days |
| PCI services available sometimes or not at all | PCI in community during work hours |
| PCI services available sometimes or not at all | PCI during hours that alternate during the week |
| PCI services available sometimes or not at all | PCI to any patient in hospital or presenting to hospital |
| PCI services available sometimes or not at all | PCI to self-presenters and patients during work hours |
| PCI services available sometimes or not at all | PCI in exceptional circumstances or never |
| PCI services available sometimes or not at all | other |

### **Additional table 9:** Baseline characteristics of people with incomplete covariate data who are dropped from the complete case analysis (N=107)

|  | **Category 1-2** | **Category 3a** | **Category 3b** | **Category 4-5** | **Total** |
| --- | --- | --- | --- | --- | --- |
|  | **N=70** | **N=21** | **N=14** | **N=<5** | **N=107** |
| **Age at index AMI hospitalisation, mean (SD)** | 67 (13) | 81 (11) | 78 (9) | 96 (4) | 71 (14) |
| **Female** | 23 (33) | 12 (57) | 7 (50) | <5 | 43 (40) |
| **Ethnicity** |  |  |  |  |  |
| White | 26 (37) | 4 (19) | 5 (36) | 0 (0) | 35 (33) |
| Other | 0 (0) | 0 (0) | 1 (7) | 0 (0) | 1 (1) |
| Missing | 44 (63) | 17 (81) | 8 (57) | <5 | 71 (66) |
| **IMD quintile** |  |  |  |  |  |
| 1 (least deprived) | 15 (21) | 6 (29) | 2 (14) | <5 | 24 (22) |
| 2 | 7 (10) | 2 (10) | 3 (21) | 0 (0) | 12 (11) |
| 3 | 13 (19) | 3 (14) | 4 (29) | 0 (0) | 20 (19) |
| 4 | 8 (11) | 5 (24) | 0 (0) | <5 | 14 (13) |
| 5 (most deprived) | 6 (9) | 2 (10) | 0 (0) | 0 (0) | 8 (7) |
| Missing | 21 (30) | 3 (14) | 5 (36) | 0 (0) | 29 (27) |
| **History of dialysis** | 0 (0) | 0 (0) | 0 (0) | 0 (0) | 0 (0) |
| **History of kidney transplant** | 0 (0) | 0 (0) | 0 (0) | 0 (0) | 0 (0) |
| **Comorbidities** |  |  |  |  |  |
| Angina | 17 (24) | 5 (24) | 3 (21) | 0 (0) | 25 (23) |
| Cerebrovascular disease | 7 (10) | 2 (10) | 3 (21) | 0 (0) | 12 (11) |
| COPD | 9 (13) | 4 (19) | 2 (14) | <5 | 16 (15) |
| Type 2 diabetes mellitus | 21 (30) | 6 (29) | 7 (50) | 0 (0) | 34 (32) |
| Heart failure | 4 (6) | 4 (19) | 2 (14) | <5 | 11 (10) |
| Hypertension | 31 (44) | 12 (57) | 12 (86) | <5 | 56 (52) |
| Myocardial infarction | 20 (29) | 7 (33) | 5 (36) | 0 (0) | 32 (30) |
| Peripheral vascular disease | 5 (7) | 1 (5) | 1 (7) | 0 (0) | 7 (7) |
| **Smoking status** |  |  |  |  |  |
| Non-smoker | 47 (67) | 13 (62) | 7 (50) | <5 | 68 (64) |
| Ever-smoker | 23 (33) | 8 (38) | 7 (50) | <5 | 39 (36) |

### **Additional table 10:** Adjusted predicted percents for dying during the index AMI hospitalisation, and for receiving angiography and/or PCI during the index AMI hospitalisation, stratified by eGFR stage

| **Outcome** | **eGFR stage** | **Adjusted predicted percent (%) (95% CI)** |
| --- | --- | --- |
| Death during index AMI hospitalisation | 1-2 | 13 (12 to 15) |
|  | 3a | 16 (14 to 18) |
|  | 3b | 19 (16 to 21) |
|  | 4-5 | 21 (17 to 25) |
| Angiography and/or PCI during index AMI hospitalisation | 1-2 | 57 (55 to 58) |
|  | 3a | 59 (56 to 61) |
|  | 3b | 51 (48 to 55) |
|  | 4-5 | 45 (40 to 50) |

### **Additional table 11:** Other outcomes during the index AMI hospitalisation and post-AMI hospitalisation.

| **Outcome** | **eGFR category** | **Events, n=** | **Rate per 100 PY (95% CI)** | **Age and sex adjusted,**  **HR (95% CI)** | **Adjusted^1^,**  **HR (95% CI)** |
| --- | --- | --- | --- | --- | --- |
| **CVD-specific death** | 1-2 | 304 | 3.74 (3.34-4.18) | 1 | 1 |
|  | 3a | 161 | 7.59 (6.51-8.86) | 1.26 (1.03-1.53) | 1.14 (0.94-1.39) |
|  | 3b | 141 | 14.37 (12.18-16.95) | 1.85 (1.49-2.28) | 1.57 (1.26-1.94) |
|  | 4-5 | 79 | 23.20 (18.61-28.93) | 3.03 (2.34-3.91) | 2.31 (1.77-3.00) |
| **AMI re-admission** | 1-2 | 316 | 22.50 (20.15-25.12) | 1 | 1 |
|  | 3a | 106 | 26.32 (21.76-31.84) | 1.00 (0.80-1.26) | 0.94 (0.74-1.18) |
|  | 3b | 44 | 21.17 (15.75-28.44) | 0.74 (0.54-1.03) | 0.66 (0.48-0.92) |
|  | 4-5 | 38 | 45.04 (32.77-61.89) | 1.41 (1.00-1.99) | 1.18 (0.83-1.68) |

^1^Adjusted for age (continuous), sex, IMD quintile, ethnicity (white or other), and history of T2DM, heart failure, COPD, and previous AMI

### **Additional table 12:** Death during the index AMI hospitalisation stratified by centre type

| **PCI services available** | **eGFR category** | **n (row %)** | **Age and sex adjusted,**  **OR (95% CI)** | **Adjusted^1^,**  **OR (95% CI)** |
| --- | --- | --- | --- | --- |
| **PCI always available** | 1-2 | 83 (12) | 1 | 1 |
|  | 3a | 62 (15) | 0.90 (0.61-1.31) | 0.84 (0.58-1.22) |
|  | 3b | 59 (23) | 1.34 (0.89-2.02) | 1.23 (0.82-1.85) |
|  | 4-5 | 36 (30) | 2.23 (1.37-3.65) | 1.78 (1.08-2.91) |
| **PCI available sometimes or not at all** | 1-2 | 316 (11) | 1 | 1 |
|  | 3a | 169 (22) | 1.51 (1.22-1.88) | 1.51 (1.21-1.89) |
|  | 3b | 124 (27) | 1.72 (1.33-2.23) | 1.66 (1.28-2.15) |
|  | 4-5 | 58 (26) | 1.84 (1.29-2.61) | 1.74 (1.23-2.45) |

^1^Adjusted for age (continuous), sex, IMD quintile, ethnicity (white or other), and history of T2DM, heart failure, COPD, and previous AMI

### **Additional table 13:** Death post-AMI hospitalisation discharge, among people who survive the first AMI hospitalisation in the study period, stratified by AMI subtype and centre type

| **PCI services available** | **eGFR category** | **Deaths, n=** | **Rate per 100 person-years** | **Age and sex adjusted,**  **HR (95% CI)** | **Adjusted^1^,**  **HR (95% CI)** |
| --- | --- | --- | --- | --- | --- |
| **PCI always available** | 1-2 | 116 | 7.16 (5.96-8.58) | 1 | 1 |
|  | 3a | 120 | 14.2 (11.9-17.0) | 1.15 (0.88-1.49) | 1.13 (0.87-1.48) |
|  | 3b | 94 | 22.9 (18.7-28.0) | 1.44 (1.08-1.93) | 1.32 (0.98-1.76) |
|  | 4-5 | 55 | 44.3 (34.0-57.7) | 3.11 (2.22-4.35) | 2.79 (1.99-3.91) |
| **PCI available sometimes or not at all** | 1-2 | 556 | 8.54 (7.86-9.28) | 1 | 1 |
|  | 3a | 239 | 18.70 (16.47-21.22) | 1.25 (1.07-1.46) | 1.13 (0.97-1.33) |
|  | 3b | 204 | 36.02 (31.40-41.32) | 1.80 (1.52-2.14) | 1.51 (1.27-1.80) |
|  | 4-5 | 128 | 59.27 (49.84-70.48) | 3.18 (2.61-3.87) | 2.56 (2.09-3.15) |

^1^Adjusted for age (continuous), sex, IMD quintile, ethnicity (white or other), and history of T2DM, heart failure, COPD, and previous AMI

### **Additional table 14:** Processes of care (angiography and/or PCI) associated with eGFR stage at baseline during the index AMI hospitalisation stratified by centre type in the overall population, and among people with STEMI hospitalisation only

| **Subgroup** | **eGFR category** | **n (row %)** | **Age and sex adjusted,**  **OR (95% CI)** | **Adjusted^1^,**  **OR (95% CI)** |
| --- | --- | --- | --- | --- |
| **PCI services available** |  |  |  |  |
| **PCI always available** | 1-2 | 581 (81) | 1 | 1 |
|  | 3a | 283 (67) | 0.83 (0.61-1.12) | 0.87 (0.64-1.19) |
|  | 3b | 134 (52) | 0.55 (0.39-0.78) | 0.62 (0.44-0.88) |
|  | 4-5 | 63 (53) | 0.43 (0.27-0.70) | 0.56 (0.35-0.90) |
| **PCI available sometimes or not at all** | 1-2 | 1,699 (59) | 1 | 1 |
|  | 3a | 319 (42) | 0.84 (0.71-1.00) | 0.91 (0.76-1.09) |
|  | 3b | 125 (27) | 0.51 (0.41-0.65) | 0.61 (0.48-0.78) |
|  | 4-5 | 54 (24) | 0.32 (0.23-0.44) | 0.38 (0.27-0.54) |
| **PCI services available (STEMI cases only)** |  |  |  |  |
| **PCI always available** | 1-2 | 315 (95) | 1 | 1 |
|  | 3a | 124 (91) | 1.38 (0.53-3.64) | 1.47 (0.59-3.65) |
|  | 3b | 53 (84) | 1.15 (0.40-3.32) | 1.29 (0.44-3.71) |
|  | 4-5 | 25 (93) | 2.04 (0.35-11.85) | 3.93 (0.69-22.53) |
| **PCI available sometimes or not at all** | 1-2 | 705 (85) | 1 | 1 |
|  | 3a | 69 (67) | 0.66 (0.41-1.05) | 0.70 (0.43-1.17) |
|  | 3b | 22 (54) | 0.50 (0.26-0.96) | 0.60 (0.29-1.23) |
|  | 4-5 | 6 (29) | 0.10 (0.035-0.29) | 0.14 (0.048-0.43) |

^1^Adjusted for age (continuous), sex, IMD quintile, ethnicity (white or other), and history of T2DM, heart failure, COPD, and previous AMI

### **Additional table 15:** Processes of care (angiography and/or PCI) during the index AMI hospitalisation restricted to people with no previous AMI hospitalisation

| **Outcome/Subgroup** | **eGFR category** | **n (row %)** | **Age and sex adjusted,**  **OR (95% CI)** | **Adjusted^1^,**  **OR (95% CI)** |
| --- | --- | --- | --- | --- |
| **Angiography and/or PCI** |  |  |  |  |
| **People with no previous AMI hospitalisation** | 1-2 | 1,893 (65) | 1 | 1 |
|  | 3a | 459 (52) | 1.04 (0.88-1.23) | 1.08 (0.91-1.28) |
|  | 3b | 194 (38) | 0.72 (0.58-0.89) | 0.83 (0.67-1.03) |
|  | 4-5 | 74 (34) | 0.44 (0.32-0.61) | 0.53 (0.38-0.73) |

### **Additional table 16:** MDRD Study equation(2)

| GFR (mL/min/1.73 m^2^) = 175 × (S_cr_)^-1.154^ × (Age)^-0.203^ × (0.742 if female) × (1.212 if African American) |
| --- |

**Additional figure 1:** Histogram describing the time between the most recent eGFR recorded in primary care (used to define baseline kidney function) and the index AMI hospitalisation
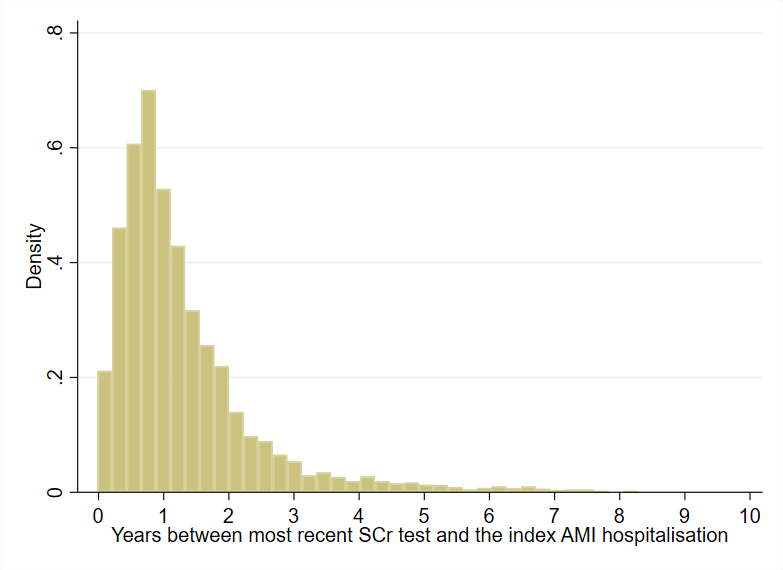


### **Additional figure 2:** Processes of care (angiography and/or PCI) associated with eGFR stage at baseline during the index AMI hospitalisation stratified by prevalent type 2 diabetes mellitus (T2DM) and prevalent heart failure status


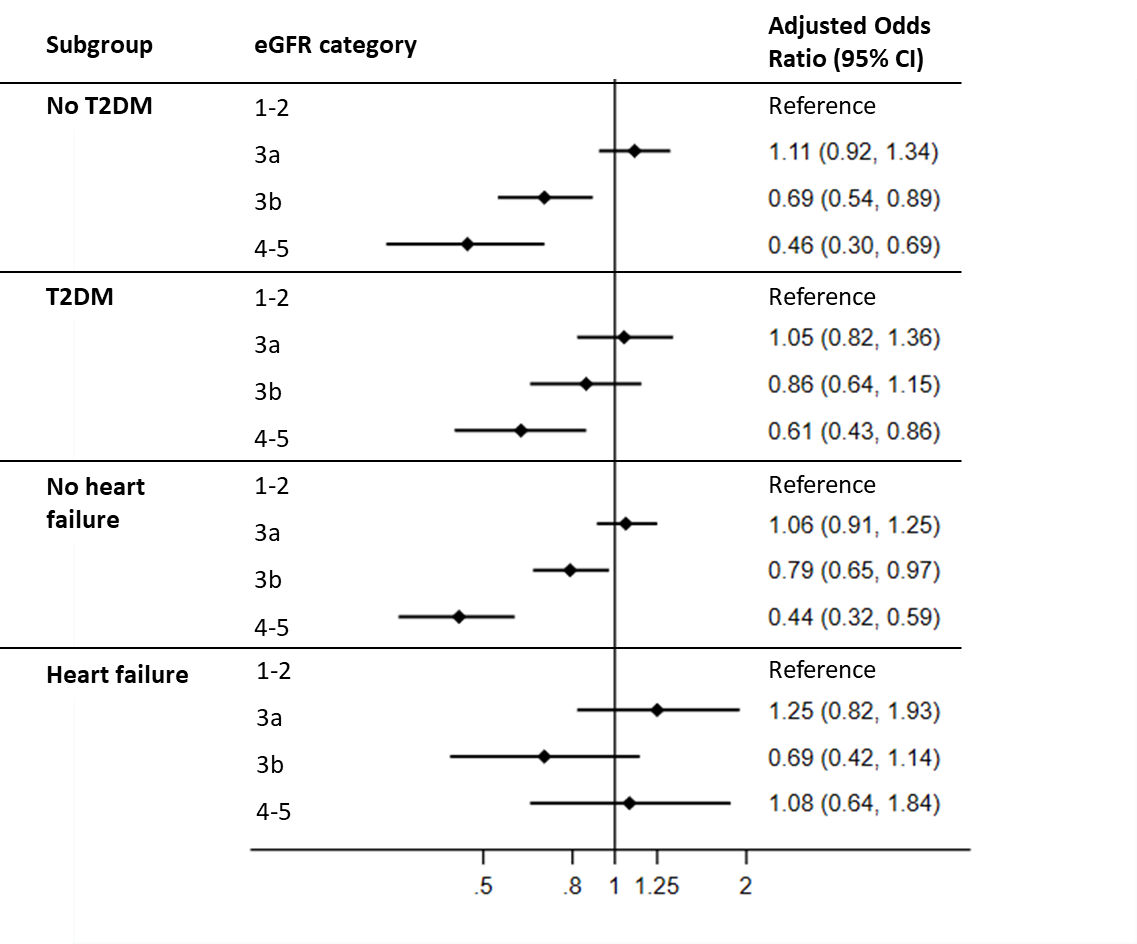


### **Additional figure 3:** In-hospital death associated with eGFR stage at baseline during the index AMI hospitalisation stratified by prevalent type 2 diabetes mellitus (T2DM) and prevalent heart failure status


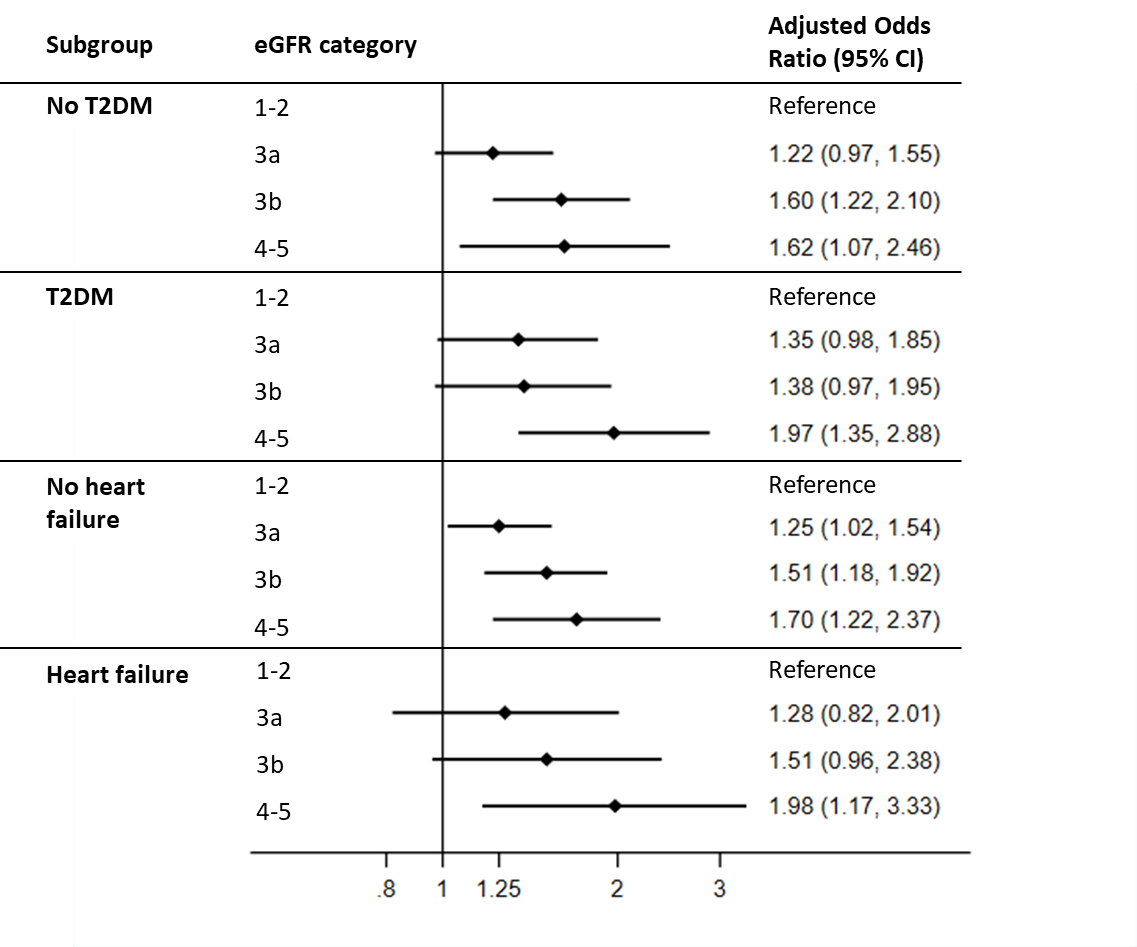


### **Additional figure 4:** Death post-AMI discharge associated with eGFR stage at baseline following the index AMI hospitalisation stratified by prevalent type 2 diabetes mellitus (T2DM) and prevalent heart failure status
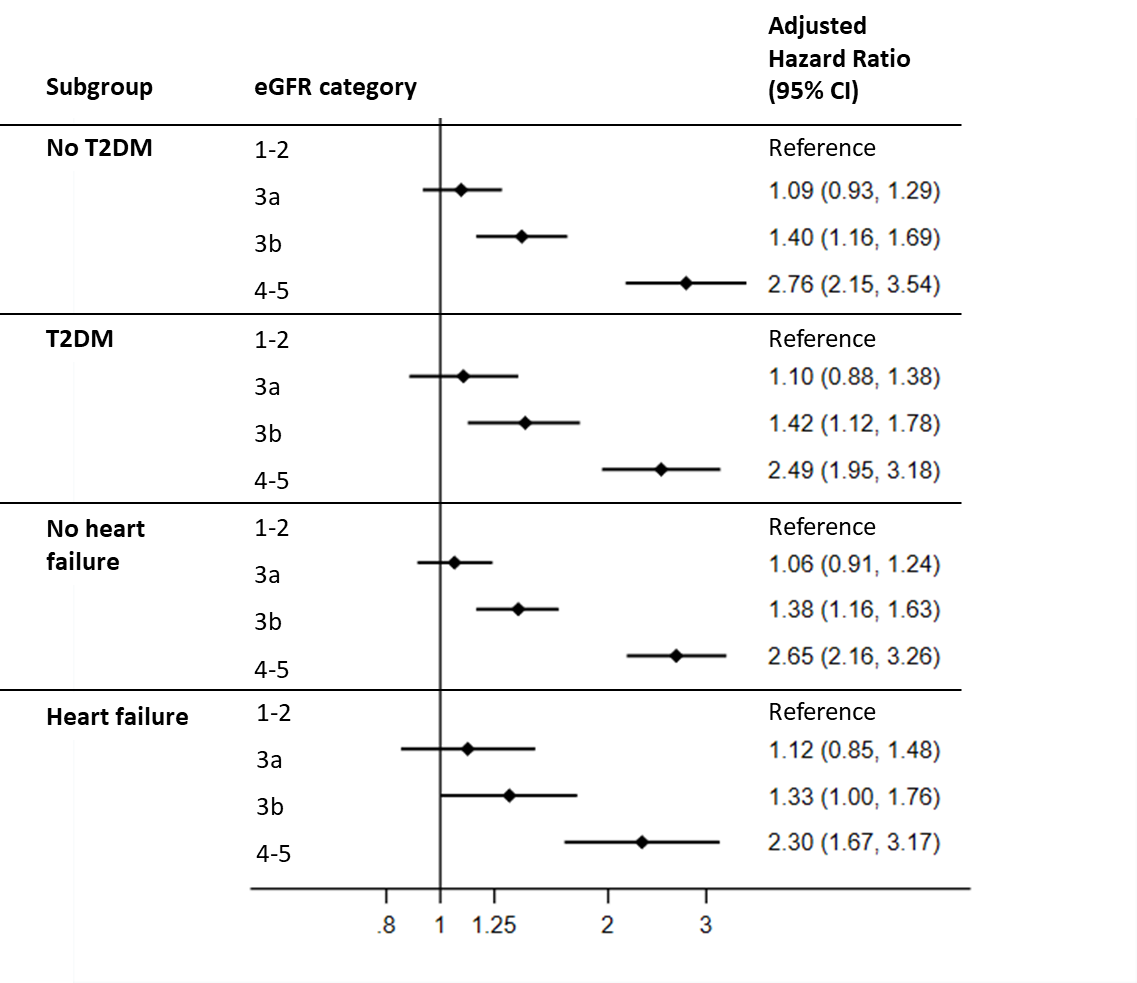


1. Nitsch D CB HS, Wheeler D. National Chronic Kidney Disease Audit - National Report Part 1. 2017.

2. Levey AS, Bosch JP, Lewis JB, Greene T, Rogers N, Roth D. A more accurate method to estimate glomerular filtration rate from serum creatinine: a new prediction equation. Modification of Diet in Renal Disease Study Group. Annals of internal medicine. 1999;130(6):461-70.
